# Increased serum resistin levels correlate with psoriasis: a meta-analysis

**DOI:** 10.1186/s12944-015-0039-9

**Published:** 2015-05-16

**Authors:** Huiyun Huang, Erdong Shen, Shiqing Tang, Xingyou Tan, Xiuli Guo, Qiang Wang, Hongwei Ding

**Affiliations:** Dermatological Department, the First People’s Hospital of YueYang, Dong Mao Ling Road No.39, YueYang, 414000 P.R. China; Department of Oncology, the First People’s Hospital of YueYang, YueYang, 414000 P.R. China

**Keywords:** Resistin, Serum level, Psoriasis, Meta-analysis

## Abstract

**Background:**

Recent studies implicate adipokines in the pathogenesis of inflammatory diseases, including psoriasis. In this study we evaluated the significance of serum resistin levels in psoriasis patients using a meta-analysis approach.223

**Methods:**

Relevant articles were retrieved by searching the following English and Chinese databases: Cochrane Library, PubMed, Springer Link, Chinese Biomedical Database (CBM) and Chinese National Knowledge Infrastructure (CNKI). The retrieved studies were subjected to a thorough screening procedure to identify case–control studies that contained the required data. Data was extracted from each study and Version 12.0 STATA statistical software was employed for statistical analyses.

**Results:**

Nine case–control studies, containing 421 psoriasis patients and 348 healthy controls, were included in this study. The major result of the meta-analysis revealed a statistically significant association between serum resistin levels and psoriasis (SMD = 2.22, 95%CI: 1.14-3.29, *P* < 0.001). Subgroup analysis based on ethnicity showed that, compared to the healthy controls, serum resistin levels were markedly higher in psoriasis patients in both Asian and Caucasian populations (Asians: SMD = 3.27, 95%CI = 1.62 ~ 4.91, *P* < 0.001; Caucasians: SMD = 0.91, 95%CI = 0.28 ~ 1.54, *P* < 0.001).

**Conclusions:**

Based on our results, we conclude that serum resistin level in psoriasis patients is higher than healthy controls, and raises the possibility that elevated serum resistin levels may be a novel diagnostic marker in psoriasis and may predict the occurrence of co-morbidities in psoriasis patients.

## Introduction

Psoriasis is one of the most common chronic inflammatory skin diseases characterized by infiltration of T cells and hyper-proliferative keratinocytes [1,2]. Psoriasis affects 2-3% of population worldwide, with equal gender distribution, and can lead to substantial morbidity and mortality directly from the disease as well as from the effect of co-morbodities [1,3]. Psoriasis has a major negative impact on quality of life in patients and considerable psychosocial disability is associated with the disease, along with the high costs of treatment [4]. Psoriasis can emerge at any time in life but it usually peaks between the ages of 30–39 and 60–69 and is associated with co-morbidities such as cardiovascular diseases, psoriatic arthritis and depression [2,5]. The prevalence rate of psoriasis around the world depends on the geographical region and ethnicity. Japan has the lowest incidence of psoriasis (0.2%), while Faroe Islands has the highest incidence (2.8%) [2,4]. Psoriasis affects both skin and the joints, ranging in severity from a few scattered red, scaly plaques to the involvement of the entire body surface [4,6]. Psoriasis patients often need life-long treatment and medical support, and the condition may progressively worsen with age or fluctuate in severity, depending on genetic and environmental factors [4,7]. Psoriasis patients are especially prone to premature atherosclerosis and increased risk of cardiovascular events [2]. The pathogenesis of psoriasis is only partly understood and involves genetic, environmental and immunological factors, with other contributing factors such as smoking, stress, trauma, and alcohol consumption [4,7]. Interestingly, psoriasis is associated with overweight and with increased serum C-reactive protein, leptin and resistin levels [5]. Recent study shows that resistin plays a predominant role in the pathogenesis of psoriasis, particularly in obese patients [8].

Resistin is a 12.5 kDa polypeptide that belongs to the inflammatory zone protein family [9,10]. Resistin is a cysteine rich protein primarily produced by peripheral blood mononuclear cells in humans and by white adipose tissues in rodents [11]. Previous studies showed that resistin regulates insulin sensitivity and glucose metabolism, and is a mediator between diabetes and obesity, exhibiting a positive correlation with body mass index [12,13]. Importantly, resistin induces the secretion of pro-inflammatory cytokines, such as interleukin-6 (IL-6), IL-12 and tumor necrosis factor α (TNF-α) [9,14]. Accordingly, elevated serum resistin level is detected in obesity and in a variety of malignancies such as colorectal cancer, breast cancer, non-small cell lung cancer and prostate cancer [15-17]. Interestingly, high serum resistin levels are also observed in psoriasis patients [18]. Therefore, it is possible that, in addition to the involvement of resistin in metabolic syndrome, resistin mediated increase in secretion of pro-inflammatory cytokines such as IL-6, IL-12 and TNF-α, through activation of nuclear factor-B signal pathway, may also directly effect the pathogenesis of psoriasis [19,20]. Consistent with this view, serum levels of resistin are positively associated with psoriasis severity, with higher serum resistin levels found in more advanced stages of psoriasis [21,22]. Therefore, resistin level may be useful as a biomarker for diagnosis of psoriasis and to adjust the treatment regimen during fluctuating severity of the disease [21]. Accordingly, multiple studies proposed that serum level of resistin may be effective for predicting the presence of psoriasis [18,23], but other studies showed contradictory results [24,25]. Therefore, in the present meta-analysis, we evaluated the relationship between serum resistin levels and psoriasis in order to test the feasibility of using serum resistin levels as diagnostic and prognostic tool for psoriasis.

## Materials and methods

### Search strategy

Published case–control studies were identified via comprehensive search (last search April 30st, 2014) of Cochrane Library, PubMed, Springer Link, Chinese Biomedical Database (CBM) and Chinese National Knowledge Infrastructure (CNKI) databases. The studies reporting the association between serum Resistin levels and psoriasis were identified from the databases by utilizing the search terms (“Resistin”), and (“Psoriasis” or “psoriasis” or “Psoriasis Vulgaris” or “Psoriasis arthropathica” or “Pustular psoriasis” or “Pustulosis Palmaris et Plantaris” or “Palmoplantaris Pustulosis” or “Pustular Psoriasis of Palms and Soles”). Additionally, manual searches were employed to identify potentially relevant articles from cross-references of important studies.

### Study selection

Randomized intervention case–control studies investigating the association between serum resistin levels and psoriasis were considered for this meta-analysis. The diagnosis of the enrolled psoriasis patients was based on the characteristic signs and symptoms and was confirmed by histopathological examination [26]. Studies with insufficient information on serum resistin levels were excluded. The included studies contained more than 50 cases per study and studies with less than 50 psoriasis cases were excluded. Duplicate studies or studies lacking complete or unavailable data were also a cause for exclusion. If the same sample was used in previous studies, only the most complete or the most recent study was enrolled.

### Data extraction

To minimize bias and improve the reliability, two investigators independently collected information based on the selection criteria and reached a consensus on all items after through discussion and reexamination. The following relevant data were extracted from the eligible studies: surname of first author, year of publication, source of publication, study type, study design, source of publication, sample size, age, gender/sex, ethnicity and country of origin, study type, detection method of serum resistin serum levels, and resistin expression levels. Data was separately extracted and grouped into Asians and Caucasians based on ethnicity. All the authors agreed with the final enrolled studies.

### Quality assessment

The methodological quality of the enrolled studies was measured by critical appraisal skill program (CASP) criteria by two investigators (http://www.casp-uk.net/). The following criteria were used: the study focuses on a clearly focused issue (CASP01); the research problem is acceptable and the research design resolve the study problem (CASP02); the cases selected in an appropriate way (CASP03); the controls recruited in an appropriate way (CASP04); the evaluation for exposure factors is precise to minimize bias (CASP05); the study controls other important confounding factors (CASP06); the study result is complete (CASP07); the study result is accurate (CASP08); the study result is reliable (CASP09); the study result can be applied to the local population (CASP10); the study result fit with other available evidence (CASP11).

### Statistical analysis

To provide quantitative evidence and minimize variance of the summary, this meta-analysis was performed by applying random-effect model or fixed-effect model. When heterogeneity existed among studies, a random-effect model was used; otherwise a fixed-effect model was utilized. The pooled standardized mean difference (SMD) with 95% confidence intervals (CI) was calculated for case versus control category of resistin serum levels by *Z* test. To investigate potential effect modification, subgroup analyses based on ethnicity and sample size were conducted. Moreover, Cochran’s Q-statistic (P < 0.05 was regarded as statistically significant) was applied to evaluate the heterogeneity among the included trials [27]. *I*^*2*^ test evaluated the possibility of heterogeneity across studies [28]. The sensitivity analysis was conducted by removing any single study at a time to assess the reliability of the results. Funnel plot was used to evaluate publication bias that might influence the validity of the estimates. The symmetry shape of the funnel plot was further confirmed by the Egger's test [29]. A *P* value of < 0.05 was considered as statistically significant. All statistical analyses were conducted with the usage of STATA software, version 12.0 (Stata Corp, College Station, TX, USA).

## Results

### Characteristics of enrolled studies

**Figure 1:**
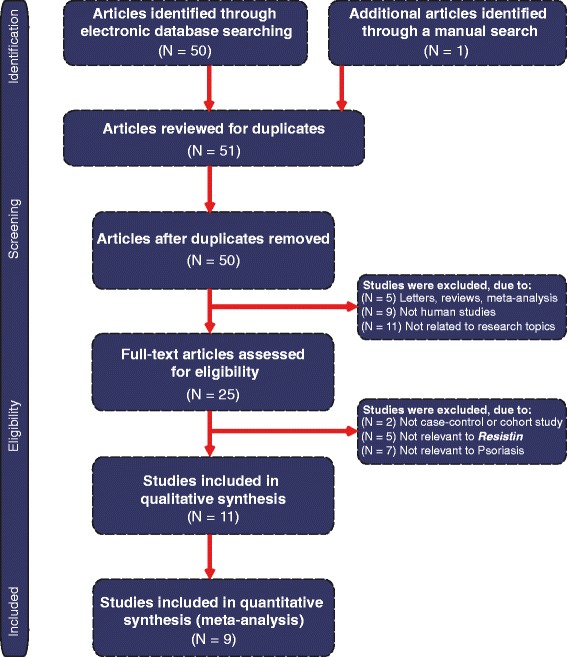
Flow chart of literature search and study selection.

**Figure 2:**
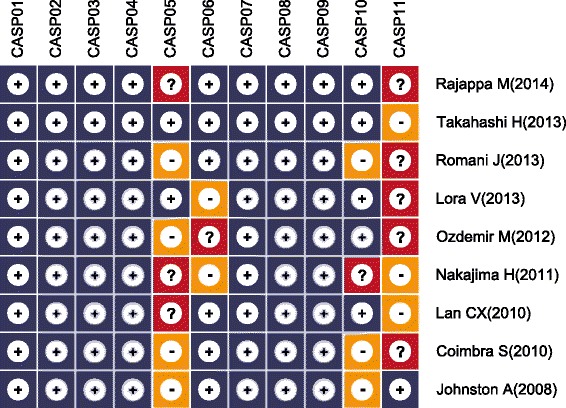
Quality of enrolled studies using critical appraisal skill program (CASP).

The database search originally resulted in retrieval of 51 articles related to the search keywords. The flow diagram of the study selection process is presented in Figure 1. After screening the title and keywords, 26 articles were removed, including 1 duplicate, 5 letters, reviews or meta-analyses, 9 non-human studies and 11 unrelated to the research topic. The remaining 25 studies were reexamined and additional 16 studies were excluded, out of which 2 was not case–control studies, 5 were not relevant to resistin, 7 were not relevant to psoriasis and 2 contained insufficient data. Finally, 9 case–control studies [18,21,23-25,30-33], containing a combined total of 769 subjects (421 patients with psoriasis and 348 healthy controls) were enrolled in this meta-analysis. The publication year of these studies ranged between 2008 and 2014. All articles were case–control studies reporting the relationship between serum resistin levels and psoriasis in Asian (5 studies) and Caucasian populations (4 studies). The detection method in all 9 studies was enzyme linked immunosorbent assay (ELISA). Table 1 displays the study characteristics and the methodological quality of the enrolled studies is shown in Figure 2.

**Table 1:** **Characteristics of included studies focused on protein expression of resistin**

**First author**	**Year**	**Ethnicity**	**Number**	**Sample size**		**Gender (M/F)**		**Age (years)**		**Method**
				**Case**	**Control**	**Case**	**Control**	**Case**	**Control**	
Rajappa M [31]	2014	Asians	Large	60	60	50/10	49/11	41.97 ± 13.40	43.75 ± 11.14	ELISA
Takahashi H [21]	2013	Asians	Large	62	58	41/21	40/18	44.2 (27~74)	39.5 (29~69)	ELISA
Romani J [24]	2013	Caucasians	Large	50	50	31/19	31/19	46.37 ± 17.29	46.06 ± 17.53	ELISA
Lora V [18]	2013	Caucasians	Small	27	27	15/12	15/12	49.9 ± 12.3	48.1 ± 8.2	ELISA
Ozdemir M [25]	2012	Asians	Small	26	26	14/12	13/13	42.4 ± 13.2	40.6 ± 10.2	ELISA
Nakajima H [23]	2011	Asians	Small	30	30	-	-	-	-	ELISA
Lan CX [30]	2010	Asians	Large	70	30	42/28	18/12	38.31 ± 3.34	37.63 ± 3.29	ELISA
Coimbra S [33]	2010	Caucasians	Large	66	37	35/31	16/21	-	-	ELISA
Johnston A [32]	2008	Caucasians	Small	30	30	16/14	13/16	52.87 (24~77)	47.14 (22~83)	ELISA

M, male; F, female; ELISA, enzyme linked immunosorbent assay; RIA, radioimmunoassay.

### Resistin serum levels in psoriasis

**Figure 3:**
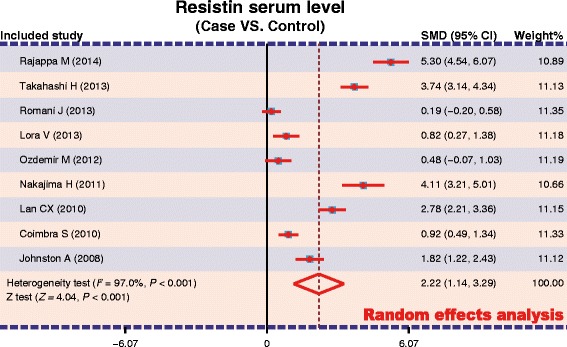
Forest plot of the differences in serum resistin levels between psoriasis and healthy controls.

**Figure 4:**
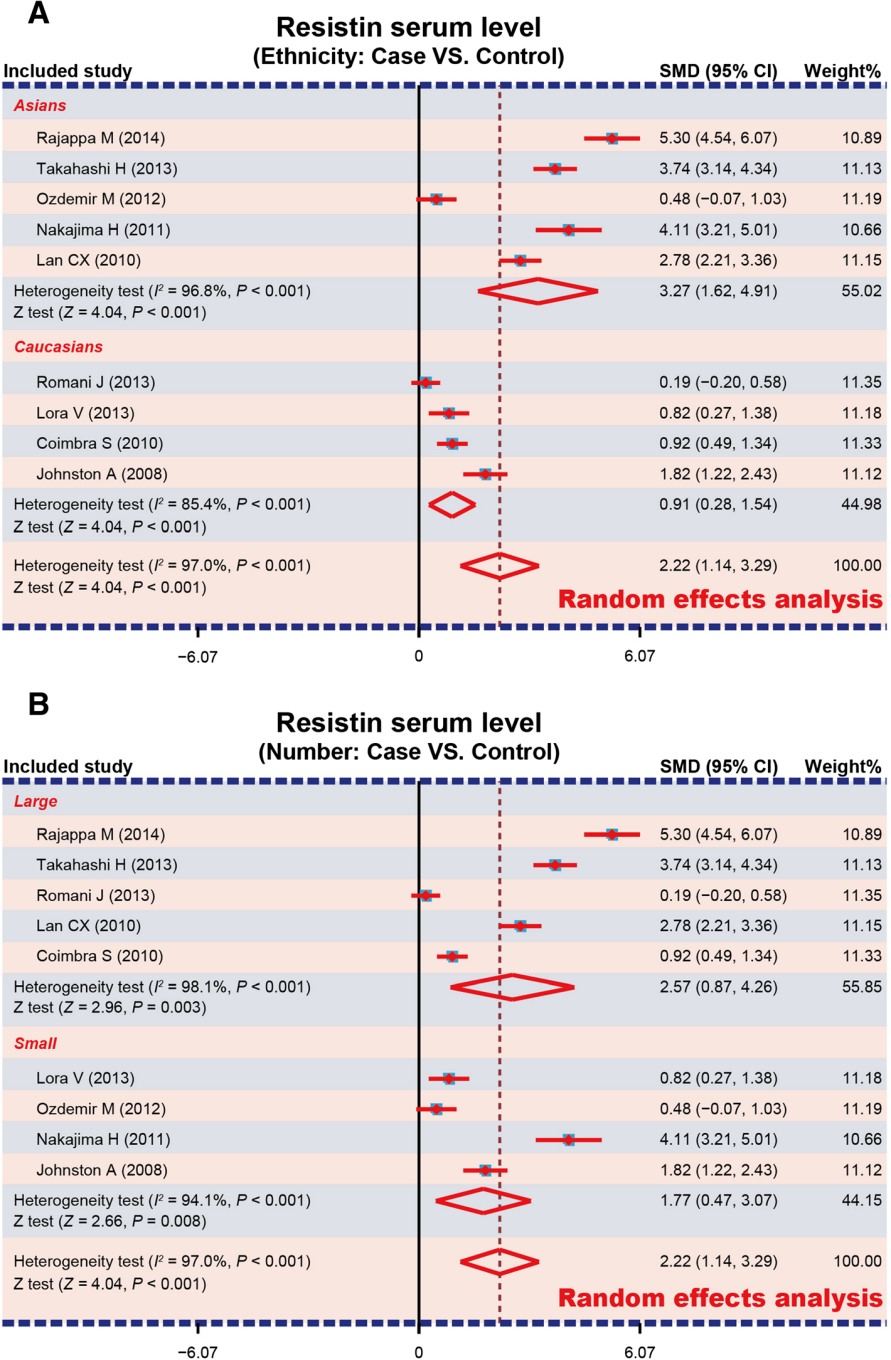
Subgroup analyses for the differences of serum resistin levels between psoriasis patients and healthy controls (**A**: Ethnicity; **B**: Number).

A total of 9 studies reported serum resistin levels in psoriasis patients. The major result of the correlation between resistin levels and psoriasis is shown in Figure 3. The random-effect model was applied due to existence of heterogeneity among the studies (*P* < 0.001). A positive association between resistin serum levels and psoriasis was identified in this meta-analysis (SMD = 2.22, 95%CI: 1.14-3.29, *P* < 0.001). Subgroup analysis based on ethnicity revealed that serum resistin levels were significantly higher in psoriasis patients, compared to healthy controls, in both Asians and Caucasians (Asians: SMD = 3.27, 95%CI = 1.62 ~ 4.91, *P* < 0.001; Caucasians: SMD = 0.91, 95%CI = 0.28 ~ 1.54, *P* < 0.001) (Figure 4A). Further, subgroup analysis by sample size indicated a statistically significant association between resistin levels and psoriasis in both large sample size and small sample size subgroups (both *P* < 0.05) (Figure 4B).

### Sensitivity analysis and publication bias

**Figure 5:**
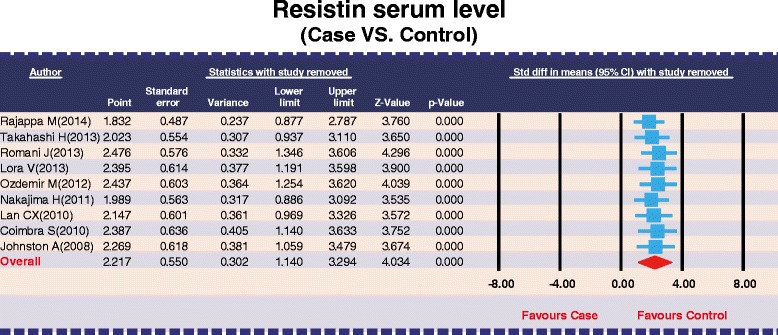
Sensitivity analysis of the summary odds ratio coefficients on the differences of serum resistin levels between psoriasis patients and healthy controls.

**Figure 6:**
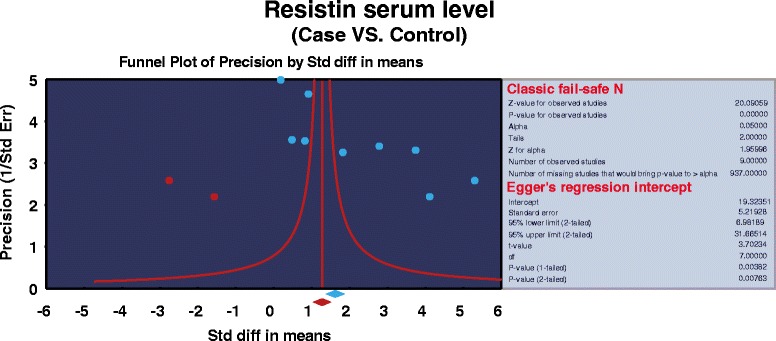
Publication biases on the differences of serum resistin levels between psoriasis patients and healthy controls.

The removal of any single study in sensitivity analysis did not change the overall statistical significance, suggesting that this meta-analysis is relatively stable and credible (Figure 5). Additionally, funnel plots of the nine studies showed slight asymmetry and Egger's test showed publication bias in our current meta-analysis (*P* = 0.008) (Figure 6).

## Discussion

In the present meta-analysis, we examined the relationship between serum levels of resistin and psoriasis. The results of our meta-analysis revealed that higher serum resistin levels positively correlated with psoriasis disease progression, indicating that resistin might be a potential biomarker for diagnosis and prognosis in psoriasis patients. The clinical features of psoriasis include epidermal hyper-proliferation, impairment of keratinocyte differentiation, increased angiogenesis and widespread immune disturbances. The dermal infiltrate in psoriasis contains various immune cells and psoriasis is largely a T-helper (Th)17/Th1-mediated autoimmune disease. More recently, a cytokine sharing the p40 unit with IL-12 and IL-23 was observed to be highly expressed in psoriatic skin. This cytokine was crucial for generation of Th17 cells with a pathogenic phenotype [19]. In humans, resistin is mainly produced by monocytes and macrophages in adipose tissue and its expression is stimulated by pro-inflammatory agents such as TNF-α, IL-1β, IL-6 and lipopolysaccharide. Resistin, in-turn can induce TNF-α and IL-12 production in activated B cells, suggesting a pathogenic cycle that sustains inflammatory cytokine production, along with resistin secretion [10,34]. Induction of CXCL8 and TNF-α by resistin in monocytes population in blood is also linked with psoriasis pathophysiology [21,35] and TNF-α stimulates keratinocyte proliferation and T cells recruitment to the skin, thus a close relationship between resistin and TNF-α-mediated inflammation exists in psoriasis [36,37]. In addition, resistin may play a role in Foxp3^+^ regulatory T-cell (Treg) deficiency in psoriasis [38]. Foxp3^+^ regulatory T-cells (Treg) are inhibitors of autoimmune responses. By inducing and maintaining immunological tolerance, Foxp3^+^ Treg cells inhibit infiltration of Foxp3^+^ lymphocytes into skin lesions in psoriasis patients [39]. Based on the above analysis, we can conclude that serum resistin level plays a pivotal role in the development of psoriasis through inflammatory factors such as TNF-α and decreased functioning of Foxp3^+^ Treg population. In line with our result, Boehncke et al. observed a close link between resistin and the PASI score, a measure for psoriasis clinical severity, implicating inflammatory mediators such as TNF-α [40].

In order to consider other factors that may affect the link between serum resistin level and psoriasis, we performed a stratified analysis based on ethnicity. Subgroup analysis showed that higher serum resistin levels were observed in both Asians and Caucasian psoriasis patients, compared to their healthy counterparts, suggesting no detectable racial differences in our analysis.

Although our meta-analysis was a practical way to generate a powerful estimate of true effect-size, with low random error compared to individual studies, several potential limitations did exist. Firstly, our study could not access all available sources that reported data related to resistin and psoriasis due to language limitations. Therefore, our study contains a relatively smaller sample size. A second limitation inherent to all meta-analysis is the possibility of publication bias, which results from publication of studies with favorable results, which is much easier than publication of unfavorable results. Another potential limitation is heterogeneity, indicating a lack of sensitivity in the pooled empirical studies. In particular, this analysis was conducted on psoriasis patients differing in clinical severity and receiving various treatments, all of which could have an influence on the results. Despite the above limitations, this is the first meta-analysis on the association between serum resistin levels and psoriasis. More importantly, our meta-analysis uses a statistical approach to combine the results from multiple studies. We avoided publication bias by using registries of trials that met our inclusion criteria. Further, inconsistent results were rigorously quantified and analyzed in our meta-analysis, which led to more reliable conclusions.

Taken together, the present study revealed higher serum resistin levels in psoriasis patients compared to healthy controls, indicating that elevated serum resistin levels strongly correlate with psoriasis progression. Therefore, serum resistin level could be a valuable biomarker in evaluating the clinical status of psoriasis patients. Nevertheless, due to the limitations in this study, it is necessary to extend our analysis to a larger population to confirm the relationship between serum resistin levels and psoriasis.
